# Women may not benefit from repeated frozen embryo transfers: a retrospective analysis of the cumulative live birth rate of 43 972 women

**DOI:** 10.1093/hropen/hoae063

**Published:** 2024-10-28

**Authors:** Yuqi Zeng, Yali Liu, Yunhan Nie, Xi Shen, Tiantian Wang, Yanping Kuang, Li Wang

**Affiliations:** Department of Assisted Reproduction, Shanghai Ninth People’s Hospital, Shanghai Jiao Tong University School of Medicine, Shanghai, PR China; Department of Assisted Reproduction, Shanghai Ninth People’s Hospital, Shanghai Jiao Tong University School of Medicine, Shanghai, PR China; Department of Assisted Reproduction, Shanghai Ninth People’s Hospital, Shanghai Jiao Tong University School of Medicine, Shanghai, PR China; Department of Assisted Reproduction, Shanghai Ninth People’s Hospital, Shanghai Jiao Tong University School of Medicine, Shanghai, PR China; Department of Assisted Reproduction, Shanghai Ninth People’s Hospital, Shanghai Jiao Tong University School of Medicine, Shanghai, PR China; Department of Assisted Reproduction, Shanghai Ninth People’s Hospital, Shanghai Jiao Tong University School of Medicine, Shanghai, PR China; Department of Assisted Reproduction, Shanghai Ninth People’s Hospital, Shanghai Jiao Tong University School of Medicine, Shanghai, PR China

**Keywords:** cumulative live birth rate (CLBR), frozen embryo transfer (FET), female age, number of oocytes, causes of infertility, ART

## Abstract

**STUDY QUESTION:**

Which specific groups of women would not benefit from repeated frozen embryo transfers (FETs)?

**SUMMARY ANSWER:**

Women over 45 years of age should stop treatment after three FET attempts due to the absence of further benefits, while women aged 40–45 years and those with a diminished ovarian reserve and other causes of infertility have a lower chance of improving their cumulative live birth rate (CLBR) within five FET cycles and experience fewer advantages from repeated transfers.

**WHAT IS KNOWN ALREADY:**

In real-life scenarios of ART, women who fail to achieve a live birth often choose to undergo repeated FETs via the freeze-all strategy.

**STUDY DESIGN, SIZE, DURATION:**

This retrospective study included 43 972 women who underwent 86 496 oocyte retrieval cycles and 82 022 FET cycles between January 2010 and March 2023 under the freeze-all strategy.

**PARTICIPANTS/MATERIALS, SETTING, METHODS:**

We categorized the population based on the female’s age at the first oocyte pick-up (OPU) cycle (Groups 1–6: <30, 30–34, 35–39, 40–42, 43–44, and ≥45 years of age), number of retrieved oocytes at the first OPU cycle (Groups 1–5: 1–5, 6–10, 11–15, 16–20, and >20 oocytes), and causes of infertility (Groups 1–9: tubal factor, male factor, polycystic ovary syndrome, diminished ovarian reserve, endometriosis, other uterine factors, combined factors, unexplained infertility, and other infertility) to analyse their CLBRs within different FET cycles via Kaplan–Meier analysis (optimistic method) and the competing risk method (conservative method). We utilized multivariate Cox and Fine–Gray models to examine the associations between the CLBR and age, the number of retrieved oocytes, and nine causes of infertility.

**MAIN RESULTS AND THE ROLE OF CHANCE:**

The CLBR decreased with increasing female age over five FET cycles (Groups 1–6: optimistic method: 96.4%, 94.2%, 86.0%, 50.2%, 23.1%, and 10.1%; conservative method: 87.1%, 82.0%, 67.8%, 33.9%, 13.8%, and 3.5%, respectively). Moreover, there was an increasing trend in the number of retrieved oocytes (Groups 1–5: optimistic method: 82.5%, 91.7%, 93.6%, 94.1%, and 96.2%; conservative method: 58.6%, 76.7%, 84.8%, 88.0%, and 92.5%, respectively). Furthermore, the CLBR varied across different causes of infertility (Groups 1–9: optimistic method: 91.7%, 93.1%, 96.6%, 79.2%, 89.9%, 76.1%, 90.0%, 92.9%, and 35.4%; conservative method: 77.3%, 79.4%, 88.9%, 46.7%, 72.7%, 62.1%, 74.4%, 78.8%, and 20.1%, respectively).

**LIMITATIONS, REASONS FOR CAUTION:**

Calculating the actual CLBR for each person is difficult because some patients have remaining embryos that have not been transferred; additionally, the current statistical methodology uses both optimistic and conservative methods to calculate the CLBR, and in real life, the CLBR falls between the optimistic and conservative curves.

**WIDER IMPLICATIONS OF THE FINDINGS:**

Our study is the first to identify specific subgroups of women who fail to benefit from repeated FETs and who require rational discontinuation of treatment following unsuccessful transfer.

**STUDY FUNDING/COMPETING INTEREST(S):**

This study was financially supported by grants from the National Natural Science Foundation of China (grant numbers: 82271732 to Y.K., 82071603 to L.W., 82001502 to Y.L., and 82201888 to X.S.). The authors declare that they have no conflicts of interest in the present study.

**TRIAL REGISTRATION NUMBER:**

N/A.

WHAT DOES THIS MEAN FOR PATIENTS? In real-life situations, women who cannot have a baby with assisted reproductive technology often choose to undergo repeated transfers (placements of embryos into the uterus) of previously frozen embryos. However, not all women benefit from this approach. It is important to identify specific groups of women who will not see any advantages from repeated frozen embryo transfers.

This study examined the total number of live births for 43 972 women who underwent 86 496 cycles for collecting eggs and 82 022 frozen embryo transfer cycles. The data are divided by age, the number of oocytes retrieved, and the reasons for infertility (grouped into nine categories). On the basis of our optimistic and conservative analyses, we discovered that it is best to stop treatment after three frozen embryo transfer attempts for women over 45 years of age because they do not benefit from this method. Women aged 40–45 years of age and those with poor ovarian function or other causes of infertility have a lower chance of improving their overall success rate within five frozen embryo transfer cycles and do not significantly increase the likelihood of a live birth from repeated transfers.

Our findings provide helpful guidance and realistic expectations for women considering multiple frozen embryo transfers after unsuccessful attempts with assisted reproductive technology.

## Introduction

With the widespread use of vitrification, freezing, and the ‘freeze-all’ strategy, frozen embryo transfer (FET) is becoming increasingly common (Maheshwari *et al.*, 2015). All embryos obtained from one or more oocyte retrieval cycles can be effectively preserved and transferred after adequate endometrium preparation, which significantly increases the live birth rate (LBR) (Zhu *et al.*, 2018). The use of this technique allows for the transfer of individual embryos until a patient achieves a successful live birth or the therapy is discontinued. Currently, the most commonly used indicator to evaluate the outcome of ART is the pregnancy rate or LBR per oocyte retrieval or transfer cycle (Wilkinson *et al.*, 2016). However, these data no longer provide a comprehensive reference for patients with repeated oocyte retrieval and embryo transfer cycles.

The cumulative live birth rate (CLBR), which refers to the proportion of deliveries resulting in at least one live birth per patient or per oocyte retrieval cycle, including all fresh and/or FETs, until one live birth is achieved or all embryos have been used, whichever occurs first, can be considered one of the most significant outcomes from the patient perspective (Toftager *et al.*, 2017; Saket *et al.*, 2021). This approach effectively addresses the limitation of the LBR in accurately reflecting the cumulative success rate of multiple embryo transfer cycles, thereby providing a more precise depiction of the actual situation (Chen *et al.*, 2015, 2022; Law *et al.*, 2019; Wang *et al.*, 2021; Zhang *et al.*, 2021). However, there is a lack of research reporting on specific subgroups of women who would not benefit from repeated FETs and would therefore require rational termination of treatment.

There were 43 972 patients who underwent 86 496 oocyte retrieval cycles and 82 022 FET cycles between 2010 and 2023 at our centre. We primarily analysed key patient demographic parameters, such as age, the number of oocytes retrieved, and the cause of infertility, and performed an in-depth investigation into the CLBR over five FET cycles within these three distinct subgroups. This significant research closely mirrors real-life scenarios and will enable various patient groups to make informed decisions regarding the continuation of therapy after unsuccessful embryo transfer.

## Materials and methods

### Study setting and patients

**Figure 1.**
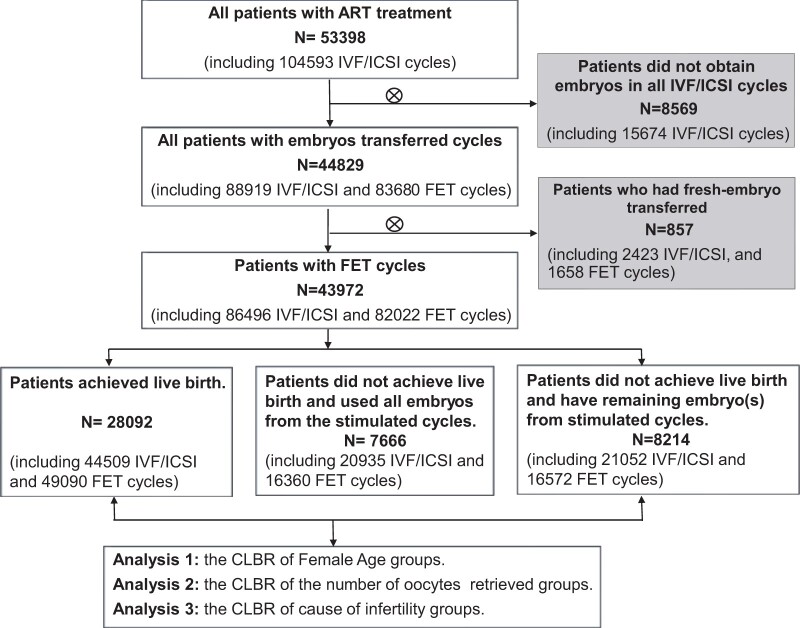
**Flow chart illustrating the study population, including the inclusion and exclusion criteria.** Patients who underwent IVF/ICSI and FET between January 2010 and March 2023 were included. Patients for whom viable embryos were not obtained in all IVF/ICSI cycles for transfer and those for whom fresh embryos were transferred were excluded. FET, frozen embryo transfer.

This retrospective cohort study was conducted in the Department of Assisted Reproduction of the Ninth People’s Hospital affiliated with the Shanghai Jiao Tong University School of Medicine. Women who received ART treatment between January 2010 and March 2023 were included. Patients for whom viable embryos for transfer were not obtained and those for whom fresh embryos were transferred were excluded. The final enrolment consisted exclusively of patients who underwent a freeze-all strategy. The CLBRs were calculated for each of the distinct groups characterized, and the flow diagram is presented in Fig. 1.

The clinical diagnosis of the nine causes of infertility was as follows: tubal factor, including fallopian tube obstruction; hydrosalpinx; history of tubal surgery; and other related factors. Male factor infertility included asthenozoospermia, oligospermia, teratozoospermia, sexual dysfunction, retrograde ejaculation, and other contributing factors. PCOS in adults was diagnosed by the Rotterdam PCOS Diagnostic Criteria (two of the following: oligo- or anovulation, clinical and/or biochemical hyperandrogenism, or polycystic ovaries shown on ultrasound) (Teede *et al.*, 2018). Diminished ovarian reserve (DOR) referred to a decrease in both the quality and quantity of oocytes retained in the ovaries. In accordance with the 2022 Chinese Expert Consensus on the Clinical Diagnosis and Treatment of DOR, we used the following criteria: an anti-Müllerian hormone concentration <1.1 ng/ml, an antral follicle count (AFC) <5–7 in both ovaries, and a basal FSH concentration ≥10 IU/l in two consecutive menstrual cycles (Tal and Seifer, 2017; Pastore *et al*., 2018; Penzias *et al.*, 2020). Endometriosis was characterized by the presence of endometrial-like tissue outside the uterus (Becker *et al.*, 2022), and the initial diagnosis is typically based on symptoms such as dysmenorrhea, chronic pelvic pain, dyspareunia, and imaging findings. Laparoscopy serves as the gold standard for the diagnosis of endometriosis. Other uterine factors included uterine adhesions, endometrial polyps, inflammation, uterine anomalies, hysteromyoma, adenomyosis, etc. Unexplained infertility was defined as apparently normal ovarian function; the presence of a fallopian tube, uterus, cervix, and pelvis; an age ≤40 years; adequate coital frequency; and apparently normal testicular function, genito-urinary anatomy, and ejaculation (Guideline Group On Unexplained Infertility *et al.*, 2023). Infertility due to combined causes referred to the presence of two or more of the above seven causes. Other infertility referred to factors that could not be accounted for by these seven causes and mainly included advanced age, a history of recurrent miscarriage, and unsuccessful IVF attempts at other medical facilities.

### Ethical approval

This retrospective study was approved by the ethics committee of Shanghai Ninth People’s Hospital, Shanghai Jiao Tong University School of Medicine (SH9H-2022-T155-1).

### Laboratory protocols

A comprehensive range of controlled ovarian stimulation protocols was incorporated for patients undergoing treatment via the freeze-all strategy. These protocols primarily included progestin-primed ovarian stimulation–related protocols but also included other protocols, such as the GnRH-agonist protocol, the GnRH-antagonist protocol, mild stimulation, and alternative approaches, such as the natural cycle and gonadotropin stimulation. Following oocyte retrieval, fertilization was performed via either conventional IVF or ICSI on the basis of the sperm quality and the response to previous fertilization attempts, as described in our previous studies (Kuang *et al.*, 2014a,b, 2015; Qin *et al.*, 2016). Following IVF/ICSI, Day 3 embryos were evaluated on the basis of Cummins’ criteria (Cummins *et al.*, 1986). On Day 5 or Day 6, blastocysts with good morphology were frozen (Gardner and Simón, 2017). The endometrial preparation protocols for FET cycles included a natural cycle, a hormone replacement cycle, a letrozole mild stimulation cycle and an HMG late stimulation cycle, as previously described (Kuang *et al.*, 2014a). In each cycle, one or two embryos were transferred with continued progestin (P) supplementation until 8 weeks of gestation if pregnancy was achieved. Each patient could undergo multiple oocyte retrieval cycles, and embryos derived from all these oocyte retrieval cycles were subjected to repeated FET procedures.

### Statistical analysis

The primary outcome was the CLBR of the patients in the different groups, including all oocyte retrieval cycles and subsequent FET cycles. Two methods were used to depict the CLBR curves. First, the Kaplan–Meier method (optimistic method), which assumes that patients who do not continue treatment have an equal chance of having a live birth as those who continue treatment, was used. The time unit utilized in the Kaplan–Meier method was each individual FET cycle. The event of interest pertained to the achievement of a live birth, whereas censoring occurred when a patient failed to achieve a live birth and discontinued further embryo transfers (terminated treatment) (McLernon *et al.*, 2016; Raz *et al.*, 2018). To calculate the cumulative live birth rate on the basis of Kaplan–Meier estimates, we subtracted the probability of not achieving a live birth at each cycle from 1, thereby obtaining the cumulative probability of a live birth occurring by that cycle. Second, the competing risk model (conservative method) presumed that some patients who discontinued treatment had no possibility of having a live birth (Stolwijk *et al.*, 1996; Raz *et al.*, 2018; Abdullah *et al.*, 2020). The time unit and event of interest in the competing risk model were consistent with those in the Kaplan–Meier method. Censoring only occurred when a patient had not achieved a live birth but still possessed available embryos and chose not to proceed with the next embryo transfer, whereas a competing event was defined as all embryos being transferred (Stolwijk *et al.*, 1996; Shen *et al.*, 2022). Censoring is different from competing events, as censoring merely prevents us from observing the target outcome event, whereas competing events prevent the occurrence of the target outcome event (Kundu *et al.*, 2003; Donoghoe and Gebski, 2017). The actual CLBR curve lies between the curve which was obtained from the estimates using the Kaplan–Meier method and the curve which was obtained from the estimates using the competing risks method. The CLBR curves were plotted against the number of FET cycles. Differences between groups were evaluated via the adjusted pairwise log-rank test.

To better analyse the effects of age, the number of oocytes retrieved, and the causes of infertility on the CLBR, multivariate regression was adopted to reduce related confounding factors via the Cox model (optimistic method) and the Fine–Gray model (conservative method). Like the Cox model, the Fine–Gray model is a regression approach that examines the effects of covariates on the cumulative incidence function in competing risk data (Fine and Gray, 1999; Austin *et al*., 2016). In the Fine–Gray model, the competing event is the same as that in the competing risk model, which is the exhaustion of all available embryos for transfer. This is because when all the embryos used for transfer have been used up, there is no chance for a live birth to occur. Therefore, completing an embryo transfer competes with achieving a live birth. It considers the exhaustion of all available embryos as a competing risk and incorporates both competing risks and covariates when assessing the cumulative incidence of live births. The analysis was performed via the ‘cmprsk’ package in R studio (R version 4.1.0, R Foundation for Statistical Computing, Vienna, Austria). The primary outcome of the regressions was cumulative live birth, and the following potential confounding factors affecting the cumulative live birth rate of patients were included in the univariate analysis: infertility duration, infertility type, basal FSH concentration, AFC, male age, BMI, number of oocyte pick-up (OPU) cycles over 1.5 years, number of FETs over 1.5 years, total oocyte number, total embryo number, number of embryos transferred, number of good-quality embryos transferred, and the treatment year. Then, three principal factors and the variables mentioned above with *P* < 0.1 were used for multivariate analysis, and adjusted hazard ratios (aHRs) with 95% CIs were calculated. The detailed results can be found in Supplementary Tables S1 and S2.

The duration of the OPU cycles was defined as the date of the last oocyte retrieval minus the date of the first oocyte retrieval plus the cycle treatment time of the first OPU cycle. If there was only one OPU cycle, the duration was equal to the cycle treatment time, which was the date of oocyte retrieval minus the start of controlled ovarian hyperstimulation (COH). The total duration of ART treatment was calculated as the time between the date of the last embryo transfer and the date of the first oocyte retrieval plus the total treatment time of the initial OPU cycle. The proportion of each COH protocol used for each patient was calculated, considering that multiple regimens were administered to each patient. The mean proportion of COH protocols was subsequently evaluated for different subgroups of patients, along with the evaluation of endometrial preparation schemes.

The statistical analyses were conducted via R studio (R version 4.1.0) and SPSS (version 25, IBM Corporation, Armonk, NY, USA). The normality of the continuous variables was tested via the Shapiro–Wilk test. Normally distributed data are expressed as the mean ± SD and were analysed via one-way ANOVA. Non-normally distributed data are presented as medians (first quartile, third quartile) and were compared via the nonparametric Kruskal–Wallis test. Categorical variables are expressed as numbers (%) and were tested via the chi-square test or Fisher’s test. *Post hoc* analyses were performed with the Bonferroni correction, and a significance level of *P* < 0.05 was considered to indicate statistical significance.

## Results

### The general features of the female patients undergoing FET cycles

The study flow chart is presented in Fig. 1. After exclusion, a total of 43 972 women underwent 86 496 OPU cycles and 82 022 FET cycles. The median age was 32 (interquartile range: 29–35) years, with a median infertility duration of 3 (interquartile range: 1–5) years and a median BMI of 21.48 (interquartile range: 19.72–23.63) kg/m^2^ (Table 1). The percentages of women who underwent one, two, three, four, and five oocyte retrieval cycles were 58.7%, 20.6%, 8.8%, 4.8%, and 7.3%, respectively. Additionally, the percentages of women who underwent one, two, three, and four FET cycles were 49.8%, 28.9%, 12.6%, and 5.1%, respectively; ultimately, only 3.6% of patients underwent five or more FETs. Supplementary Tables S3 and S4 present the precise figures encompassing the entire cohort of 43 972 patients within each cycle number (time), the number of women in the treatment group (risk group), the number of live births (events), the number of women who discontinued treatment (censoring), the probability of not achieving a live birth up to each cycle, and the cumulative incidence of live births up to each FET cycle via the Kaplan–Meier method (optimistic method) and the competing risk model (conservative method) respectively. Supplementary Fig. S1 demonstrates the CLBR estimates obtained from both the Kaplan–Meier method and the competing risk method for the entire cohort of 43 972 individuals.

**Table 1.** The overall ART profiles of 43 972 female patients.

**Number of women**	43 972
**Female age (years)**	32 (29, 35)
**Duration of infertility (years)**	3 (1, 5)
**Infertility type (n, %)**	
Primary infertility	23 306 (53.00%)
Secondary infertility	20 666 (47.00%)
**Body mass index (kg/m^2^)**	21.48 (19.72, 23.63)
**Number of IVF/ICSI cycles**	86 496
IVF	49 248 (56.94%)
ICSI	28 795 (33.29%)
IVF+ICSI	6460 (7.47%)
Unknown	1993 (2.30%)
**Number of women having different oocyte retrieval cycles**	
1	25 788 (58.65%)
2	9046 (20.57%)
3	3856 (8.77%)
4	2090 (4.75%)
≥5	3192 (7.26%)
**Number of FET cycles**	82 022
**Number of women having different FET cycles**	
1	21 890 (49.78%)
2	12 727 (28.94%)
3	5560 (12.64%)
4	2225 (5.06%)
≥5	1570 (3.57%)
**Median time duration of women having different FET cycles (months)**	
1	3.83 (2.50, 6.09)
2	8.63 (5.53, 15.47)
3	16.10 (10.49, 28.05)
4	23.60 (16.07, 38.34)
≥5	37.31 (25.98, 53.93)

Numbers are mean (interquartile range, IQR) for continuous parameters and n (%) for categorical parameters. Median time duration of women having different FET cycles, the average duration between the date of the last embryo transfer and the date of the first oocyte retrieval, plus the cycle treatment time for the initial oocyte pick-up cycle.

FET, frozen embryo transfer.

### Cumulative live birth rates of different age groups

As shown in Table 2, patients were categorized into six groups according to their age at the first OPU cycle: <30 (29.3%), 30–34 (40.4%), 35–39 (20.9%), 40–42 (5.8%), 43–44 (2.7%), and ≥45 (1.5%) years. Patients in Group 6 had the highest number of oocyte retrieval cycles, and the lowest number of oocytes retrieved per OPU cycle, as well as the fewest numbers of viable embryos and good-quality embryos per OPU cycle. Additionally, patients in Groups 5 and 6 had longer infertility, OPU cycle, and ART durations.

**Table 2.** The characteristics of IVF/ICSI and frozen embryo transfer cycles in women, whose cumulative live birth rates are calculated based on different age groups and the number of oocytes retrieved.

	Age groups						Number of oocytes retrieved groups				
	Group 1	Group 2	Group 3	Group 4	Group 5	Group 6	Group 1	Group 2	Group 3	Group 4	Group 5
	<30	30–34	35–39	40–42	43–44	≥45	0–5	6–10	11–15	16–20	>20
**Number of women**	12 861 (29.25%)	17 772 (40.42%)	9192 (20.90%)	2557 (5.82%)	953 (2.17%)	637 (1.45%)	13 845 (31.49%)	11 718 (26.65%)	8662 (19.70%)	5041 (11.46%)	4706 (10.70%)
**Age (years)**	28 (26, 29)^a^	32 (21, 33)^b^	36 (35, 38)^c^	41 (40, 42)^d^	43 (43, 44)^e^	46 (45, 47)^f^	35 (31, 39)^a^	32 (29, 35)^b^	31 (28, 34)^c^	30 (28, 33)^d^	29 (27, 32)^e^
**Body mass index (kg/m^2^)**	21.16 (19.47, 23.44)^a^	21.36 (19.63, 23.44)^b^	21.72 (20.03, 23.73)^c^	22.22 (20.55, 24.05)^d^	22.31 (20.81, 24.22)^d,e^	22.68 (21.09, 24.61)^e^	21.80 (20.03, 24.03)^a^	21.45 (19.71, 23.50)^b^	21.30 (19.56, 23.44)^b^	21.30 (19.53, 23.44)^b^	21.26 (19.53, 23.44)^b^
**Infertility duration (years)**	2 (1, 4)^a^	3 (2, 5)^b^	3 (1, 6)^c^	3 (1, 7)^c,d^	2 (1, 6)^a^	2 (1, 5)^a^	3 (1, 5)^a^	3 (1, 5)^b^	3 (1, 5)^c^	3 (1, 4)^b,c^	3 (1, 4)^c^
**OPU cycles per woman (n)**	1 (1, 2)^a^	1 (1, 2)^b^	2 (1, 3)^c^	2 (1, 4)^d^	3 (2, 5)^e^	3 (2, 6)^e^	2 (2, 4)^a^	1 (1, 2)^b^	1 (1, 1)^c^	1 (1, 1)^d^	1 (1, 1)^e^
**Time duration of total OPU cycles (months)**	0.40 (0.37, 1.79)^a^	0.40 (1.37, 3.78)^b^	1.03 (0.37, 7.33)^c^	4.00 (0.47, 11.67)^d^	5.83 (1.56, 14.57)^e^	5.85 (2.21, 12.10)^e^	3.60 (1.03, 10.57)^a^	0.40 (0.37, 4.37)^b^	0.37 (0.33, 0.50)^c^	0.37 (0.33, 0.43)^c^	0.37 (0.37, 0.40)^d^
**COH protocol**	a	b	c	d	e	f	a	b	c	c	d
PPOS related (n, %)	12 239 (64.04%)	19 212 (61.56%)	12 494 (59.53%)	4871 (57.48%)	2340 (58.14%)	1623 (60.36%)	25 285 (57.73%)	12 220 (62.62%)	7540 (64.62%)	4073 (65.69%)	3661 (68.88%)
Mild stimulation (n, %)	2141 (11.20%)	3793 (12.16%)	2920 (13.91%)	1357 (16.01%)	639 (15.88%)	410 (15.25%)	7604 (17.36%)	2021 (10.36%)	873 (7.48%)	415 (6.69%)	347 (6.53%)
GnRH agonist (n, %)	2305 (12.06%)	3813 (12.22%)	2082 (9.92%)	550 (6.49%)	148 (3.68%)	49 (1.82%)	2756 (6.29%)	2803 (14.36%)	1823 (15.62%)	928 (14.97%)	637 (11.99%)
GnRH antagonist (n, %)	1723 (9.02%)	2944 (9.43%)	1894 (9.02%)	764 (9.02%)	325 (8.07%)	179 (6.66%)	3361 (7.67%)	1989 (10.19%)	1192 (10.21%)	690 (11.13%)	597 (11.23%)
Others (n, %)	704 (3.68%)	1446 (4.63%)	1598 (7.61%)	932 (11.00%)	573 (14.23%)	428 (15.92%)	4792 (10.94%)	481 (2.47%)	241 (2.07%)	94 (1.52%)	73 (1.37%)
**Oocytes retrieved per OPU (n)**	11 (6, 17)^a^	7 (3, 13)^b^	4 (2, 8)^c^	2 (1, 5)^d^	2 (1, 3)^e^	1 (1, 3)^f^	2 (1, 4)^e^	8 (6, 9)^d^	12 (11, 14)^c^	17 (16, 19)^b^	24 (22, 29)^a^
**Viable embryos per OPU (n)**	4 (2, 6)^a^	2 (1, 5)^b^	2 (1, 3)^c^	1 (0, 2)^d^	1 (0, 2)^e^	1 (0, 1)^f^	1 (0, 2)^e^	3 (2, 4)^d^	4 (3, 6)^c^	6 (4, 8)^b^	8 (5, 10)^a^
**Good embryos per OPU (n)**	3 (1, 6)^a^	2 (1, 4)^b^	1 (0, 3)^c^	1 (0, 2)^d^	1 (0, 1)^e^	1 (0, 1)^f^	1 (0, 2)^e^	2 (1, 4)^d^	4 (2, 6)^c^	5 (2, 8)^b^	7 (4, 11)^a^
**FET cycles per women (n)**	1 (1, 2)^a,b^	1 (1, 2)^a^	2 (1, 2)^b^	2 (1, 3)^c^	2 (1, 3)^c^	2 (1, 2)^a,b,c^	1 (1, 2)^a^	2 (1, 2)^b^	2 (1, 2)^c^	2 (1, 2)^c^	2 (1, 2)^c^
**Transferred embryos per FET cycle (n)**	2 (1, 2)^a^	2 (1, 2)^a^	2 (1, 2)^a^	2 (1, 2)^a^	2 (1, 2)^a^	2 (1, 2)^a^	2 (1, 2)^a^	2 (1, 2)^b^	2 (1, 2)^a,b^	2 (1, 2)^a,b^	2 (1, 2)^a,b^
**Total transferred embryos per women (n)**	2 (2, 4)^a,b^	2 (2, 4)^a^	2 (2, 4)^b^	3 (2, 4)^c^	3 (2, 5)^c^	2 (2, 4)^a,b^	2 (2, 4)^a^	2 (2, 4)^b^	3 (2, 4)^c^	3 (2, 4)^c,d^	3 (2, 4)^d^
Cleavage embryos (n)	2 (2, 4)^a^	2 (2, 4)^a^	2 (2, 4)^b^	2 (2, 4)^c^	3 (2, 4)^d^	2 (1, 4)^e^	2 (2, 4)^a^	2 (2, 4)^b^	2 (2, 4)^c^	2 (2, 4)^d^	2 (2, 4)^e^
Blast embryos (n)	0 (0, 1)^a^	0 (0, 1)^a^	0 (0, 1)^b^	0 (0, 1)^c^	0 (0, 0)^d^	0 (0, 0)^e^	0 (0, 0)^a^	0 (0, 1)^b^	0 (0, 1)^c^	0 (0, 1)^d^	0 (0, 1)^e^
**Endometrium preparation protocol**	a	b	c	d	e	f	a	b	c	c	d
Natural cycles (n, %)	4007 (17.00%)	6690 (20.52%)	3839 (22.17%)	1081 (20.84%)	425 (21.05%)	235 (19.88%)	5388 (21.50%)	4779 (22.22%)	3231 (19.60%)	1699 (17.40%)	1180 (13.04%)
Hormone replacement (n, %)	8150 (34.59%)	11 588 (35.55%)	6753 (39.00%)	2368 (45.64%)	1036 (51.31%)	682 (57.70%)	10 964 (43.75%)	7349 (34.16%)	5521 (33.49%)	3389 (34.71%)	3354 (37.08%)
Letrozole mild stimulation (n, %)	7160 (30.39%)	8501 (26.08%)	3554 (20.52%)	761 (14.67%)	250 (12.38%)	105 8.88%)	4246 (16.94%)	5160 (23.99%)	4635 (28.11%)	3005 (30.77%)	3285 (36.31%)
HMG late stimulation (n, %)	4247 (18.02%)	5819 (17.85%)	3171 (18.31%)	978 (18.85%)	308 (15.26%)	160 (13.54%)	4462 (17.81%)	4223 (19.63%)	3099 (18.80%)	1672 (17.12%)	1227 (13.56%)
**Time duration of total ART treatment (months)**	5.73 (3.20, 13.23)^a^	6.20 (3.43, 13.17)^b^	7.57 (4.07, 15.47)^c^	10.07 (5.14, 18.71)^d^	11.47 (6.23, 20.29)^e^	10.59 (5.97, 18.41)^d,e^	8.93 (4.80, 17.56)^a^	6.07 (3.43, 13.17)^b^	5.63 (3.23, 12.60)^c^	5.50 (3.10, 12.47)^c,d^	5.25 (2.93, 11.94)^d^

Non-normally distributed data are presented as medians (first quartile, third quartile) and compared via nonparametric Kruskal–Wallis test. Categorical variables are expressed as numbers (%) and tested using chi-square test or Fisher’s test. *Post hoc* analyses were performed with the Bonferroni test. Different letters a, b, c, d, e and f represent significant differences between groups: *P* < 0.05.

CLBR, cumulative live birth rate; OPU, oocyte pick-up; COH, controlled ovarian hyperstimulation; PPOS, progestin-primed ovarian stimulation; FET, frozen embryo transfer.

**Figure 2.**
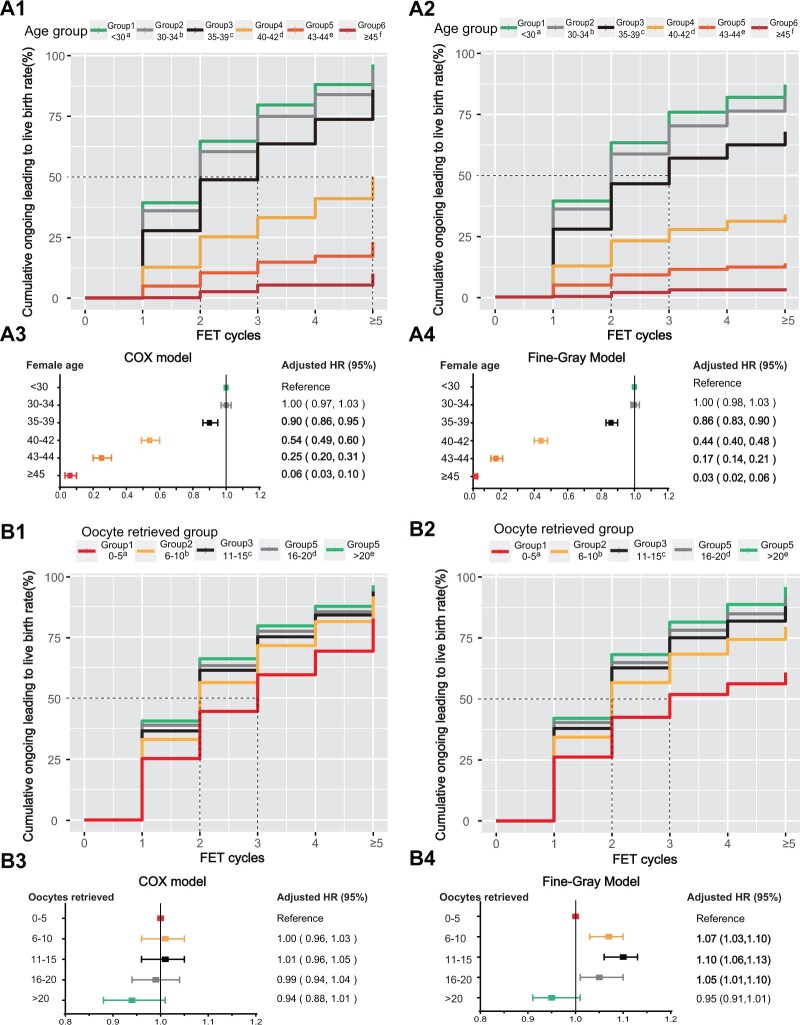
**Cumulative live birth curves for patients who underwent more than five frozen embryo transfer cycles stratified by female age and the number of oocytes retrieved.** (**A**) Female patients were categorized on the basis of their age at the first oocyte retrieval cycle. The cumulative live birth rate was calculated via Kaplan–Meier analysis (optimistic method) (**A1**) and the competing risk method (conservative method) (**A2**). The adjusted hazard ratios (HRs) with 95% CI of the cumulative live birth rates determined via the Cox model (**A3**) and Fine-Gray model (**A4**) for each group are presented below the cumulative live birth rate curves. (**B**) Female patients were stratified according to the number of oocytes retrieved at their first oocyte retrieval cycle. The cumulative live birth rate was calculated via Kaplan–Meier analysis (the optimistic method) (**B1**) and the competing risk method (the conservative method) (**B2**). The adjusted HRs with 95% CI of the cumulative live birth rates were determined via the Cox model (**B3**) and Fine–Gray model (**B4**). The distinct letters a, b, c, d, and e above the group legends for both the optimistic and conservative methods indicate statistically significant differences between groups. All curves exhibited significant differences among these five groups (*P* < 0.001), as determined by pairwise log-rank test analysis. FET, frozen embryo transfer.

The CLBRs after the first FET cycle were 39.3%, 36.0%, 27.8%, 12.7%, 4.8%, and 0.2% for Groups 1–6, respectively, for both the optimistic and conservative methods (Fig. 2A1 and A2). When the number of FET cycles was equal to or greater than five, these percentages increased to 96.4%, 94.2%, 86.0%, 50.2%, 23.1%, and 10.1% with the optimistic method (Fig. 2A1) and 87.1%, 82.0%, 67.8%, 33.9%, 13.8%, and 3.5% with the conservative method (Fig. 2A2). After adjusting for confounding factors, a decreasing trend in the aHR was observed among the five age groups (Fig. 2A3 and A4).

**Figure 3.**
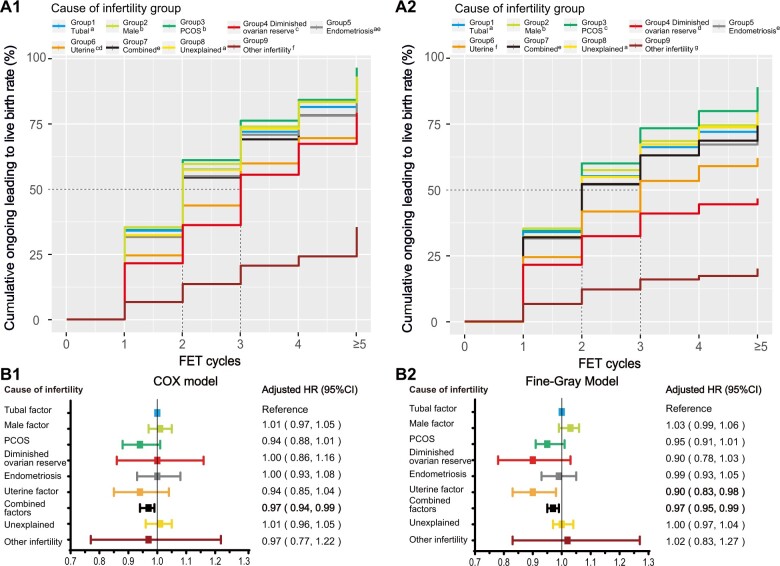
**Cumulative live birth curves for patients who underwent more than five frozen embryo transfer cycles stratified by cause of infertility.** (**A**) The cumulative live birth rates of the groups with different causes of infertility were calculated via Kaplan–Meier analysis (the optimistic method) (**A1**), and the competing risk method (conservative method) (**A2**). (**B**) The adjusted hazard ratios (HRs) of the cumulative live birth rates were computed via the Cox model (**B1**) and the Fine–Gray model (**B2**). The results of pairwise log-rank comparisons among the eight groups are indicated in the figure. A significance level of *P* < 0.05 was considered to indicate statistical significance. The distinct letters a, b, c, d, e, f, and g above the group legends indicate statistically significant differences between groups.

### Cumulative live birth rates of the groups with different numbers of oocytes retrieved

As shown in Table 2, patients were categorized into Groups 1–5 based on the number of oocytes retrieved during the first OPU cycle: 0–5 (31.5%), 6–10 (26.7%), 11–15 (19.7%), 16–20 (11.5%), and ≥20 (10.7%) oocytes. Group 1 had the longest duration of infertility, underwent the greatest number of oocyte retrieval cycles, and experienced the longest OPU cycle duration. Patients in this group had the lowest number of oocytes retrieved per OPU cycle and had the fewest numbers of viable embryos and good-quality embryos.

The CLBRs after the first FET cycle for the groups with different numbers of retrieved oocytes were 25.2%, 33.0%, 36.5%, 38.9%, and 40.6% for Groups 1–5, respectively, for both the optimistic and conservative methods (Fig. 2B1 and B2). Furthermore, the CLBRs over five FET cycles were 82.5%, 91.7%, 93.6%, 94.1%, and 96.2% for Groups 1–5, respectively, when the optimistic method was used (Fig. 2B1). Conversely, employing the conservative method yielded CLBRs of 58.61%, 76.7%, 84.8%, 88.0%, and 92.5% for Groups 1–5, respectively (Fig. 2B2). According to the multivariate regression analysis, the Cox model revealed no significant difference among the groups after stratification by the number of oocytes retrieved. However, in the Fine–Gray model, Groups 2–4 showed an increasing trend for the aHR compared with Group 1.

### Cumulative live birth rates among the different groups categorized by the cause of infertility

As shown in Table 3, the predominant cause of infertility was tubal factor infertility (41.6%), followed by combined factors (28.0%), male factor infertility (11.3%), unexplained infertility (9.2%), PCOS (3.0%), endometriosis (2.6%), uterine factor infertility (1.8%), other infertility (1.4%), and DOR (1.1%). The PCOS group exhibited superior performance in terms of the number of oocyte retrieval cycles, despite having the longest duration of infertility and the highest BMI. Patients with other infertility causes were the oldest (43 years old, interquartile range: 42–45), had the greatest number of OPU cycles and had the greatest number of embryos transferred.

**Table 3.** The characteristics of IVF/ICSI and frozen embryo transfer cycles in women, whose cumulative live birth rates are calculated based on different causes of infertility.

	Causes of infertility groups								
	Group 1	Group 2	Group 3	Group 4	Group 5	Group 6	Group 7	Group 8	Group 9
	Tubal factor	Male factor	PCOS	DOR	Endometriosis	Uterine factor	Combined factors	Unexplained	Other infertility
**Number of women (n)**	18 303 (41.62%)	4965 (11.29%)	1316 (2.99%)	488 (1.11%)	1130 (2.57%)	782 (1.78%)	12 309 (27.99%)	4063 (9.24%)	616 (1.40%)
**Age (years)**	32 (29.350)^a^	31 (28, 35)^b^	30 (28, 32)^c^	35 (32, 40)^d^	32 (30, 35)^a^	34 (31, 38)^e^	32 (29, 35)^a^	32 (29, 35)^a^	43 (42, 45)^f^
**Body mass index (kg/m^2^)**	21.36 (19.63, 23.44)^a^	21.26 (19.53, 23.44)^a^	24.03 (21.33, 27.01)^b^	21.48 (19.89, 23.58)^a^	20.57 (19.15, 22.58)^c^	21.60 (19.63, 23.44)^a^	21.63 (19.84, 24.01)^a,d^	21.45 (19.60, 23.46)^a^	22.32 (20.83, 24.03)^e^
**Infertility duration (year)**	3 (1, 5)^a^	3 (2, 5)^a^	3 (2, 5)^b^	2 (1, 4)^c^	3 (1, 5)^a,c^	2 (1, 4)^a,b,c^	3 (2, 5)^a,b^	3 (2, 5)^a^	2 (0, 4)^a,c^
**OPU cycles per woman (n)**	1 (1, 2)^a^	1 (1, 2)^b^	1 (1, 1)^c^	3 (2, 5)^d^	2 (1, 3)^e^	1 (1, 2)^f^	1 (1, 2)^f,g^	1 (1, 2)^a,h^	3 (2, 5)^d,i^
**Time duration of total OPU cycles (month)**	0.43 (0.37, 4.47)^a^	0.40 (0.37, 3.43)^b^	0.40 (0.37, 0.67)^b,c^	4.53 (1.23, 12.53)^d^	1.42 (0.37, 6.42)^e^	0.47 (0.37, 5.38)^e^	0.43 (0.37, 5.93)^e,f^	0.40 (0.37, 3.73)^a,b^	4.25 (1.17, 11.56)^d^
**COH protocol proportion**	a	b	c	d	a	e	f	g	d
PPOS related (n, %)	19 970 (58.10%)	5234 (61.75%)	1185 (66.09%)	1164 (63.43%)	1652 (61.14%)	1089 (67.26%)	16 494 (62.98%)	4547 (63.20%)	1444 (62.40%)
Mild stimulation (n, %)	5002 (14.55%)	1016 (11.99%)	261 (14.56%)	143 (7.79%)	370 (13.69%)	144 (8.89%)	3327 (12.70%)	737 (10.24%)	260 (11.24%)
GnRH agonist (n, %)	4383 (12.75%)	933 (11.00%)	89 (4.96%)	55 (3.00%)	262 (9.70%)	124 (7.66%)	2371 (9.05%)	659 (9.16%)	71 (3.07%)
GnRH antagonist (n, %)	2932 (8.53%)	944 (11.14%)	216 (12.05%)	158 (8.61%)	186 (6.88%)	165 (10.19%)	2087 (7.97%)	872 (12.12%)	269 (11.62%)
Others (n, %)	2084 (6.06%)	349 (4.12%)	42 (2.34%)	315 (17.17%)	232 (8.59%)	97 (5.99%)	1912 (7.30%)	380 (5.28%)	270 (11.67%)
**Oocytes retrieved per OPU (n)**	6 (3, 12)^d^	8 (3, 14)^b^	13 (7, 20)^a^	1 (1, 3)^h^	4 (2, 8)^f^	5 (2, 11)^e^	5 (2, 10)^e^	7 (3, 13)^c^	2 (1, 4)^g^
**Viable embryos per OPU (n)**	2 (1, 5)^c^	3 (1, 5)^b^	4 (2, 7)^a^	1 (0, 1)^g^	2 (1, 3)^e^	2 (1, 4)^d^	2 (1, 4)^d^	2 (1, 5)^c^	1 (0, 2)^f^
**Good embryos per OPU (n)**	2 (1, 4)^a,b^	2 (1, 4)^c^	4 (1, 7)^d^	1 (0, 1)^e^	1 (0, 3)^f^	2 (1, 3)^a^	1 (0, 3)^g^	2 (1, 4)^b^	1 (0, 2)^h^
**FET cycles per women (n)**	2 (1, 2)^a^	1 (1, 2)^b^	1 (1, 2)^c^	1 (1, 2)^d^	1 (1, 2)^a,b,c^	2 (1, 2)^a^	2 (1, 2)^a^	1 (1, 2)^b,c,e^	2 (1, 2)^a,b,c^
**Transferred embryos per FET cycle (n)**	2 (1, 2)^a,b^	2 (1, 2)^a,b^	2 (1, 2)^a,b^	2 (1, 2)^a,b^	2 (1, 2)^a,b^	2 (1, 2)^a^	2 (1, 2)^b^	2 (1, 2)^b^	2 (1, 2)^b^
**Total transferred embryos per women (n)**	2 (2, 4)^a^	2 (2, 4)^b^	2 (2, 4)^a,b^	2 (1, 3)^c^	2 (2, 4)^a,b^	3 (2, 4)^a,b^	2 (2, 4)^a,b^	2 (2, 4)^b,d^	3 (2, 4)^a,b^
Cleavage embryos (n, %)	2 (2, 4)^a^	2 (2, 4)^a^	2 (1, 4)^b^	2 (1, 3)^c^	2 (2, 4)^a^	2 (2, 4)^a,d^	2 (2, 4)^a^	2 (2, 3)^d^	2 (2, 4)^e^
Blast embryos (n, %)	0 (0, 1)^a^	0 (0, 1)^a^	0 (0, 1)^b^	0 (0, 0)^c^	0 (0, 1)^a^	0 (0, 1)^a,d^	0 (0, 1)^a^	0 (0, 1)^d^	0 (0, 0)^e^
**Endometrium preparation protocol proportion**	a	b	c	d	e	f,g	f	g	h
Natural cycles (n, %)	7890 (22.84%)	1897 (21.10%)	37 (1.60%)	123 (16.06%)	483 (23.80%)	239 (15.35%)	4297 (18.39%)	1132 (19.53%)	179 (15.03%)
Hormone replacement (n, %)	12 251 (35.47%)	2925 (32.53%)	1170 (50.45%)	420 (54.83%)	828 (40.81%)	624 (40.08%)	9030 (38.64%)	2705 (38.07%)	624 (52.39%)
Letrozole mild stimulation (n, %)	7964 (23.06%)	2522 (28.05%)	931 (40.15%)	84 (10.97%)	353 (17.40%)	386 (24.79%)	5892 (25.21%)	2039 (28.70%)	160 (13.43%)
HMG late stimulation (n, %)	6434 (18.63%)	1647 (18.32%)	181 (7.81%)	139 (18.15%)	365 (17.99%)	308 (19.78%)	4152 (17.76%)	1229 (17.30%)	228 (19.14%)
**Time duration of total ART treatment (month）**	6.63 (3.63, 14.73)^a^	5.53 (3.02, 12.43)^b^	5.14 (3.00, 10.73)^b,c^	9.15 (4.77, 16.55)^d^	7.50 (4.19, 15.57)^d,e^	6.95 (3.79, 14.63)^a,e^	7.30 (3.95, 16.02)^e^	5.43 (3.13, 11.33)^b,f^	9.50 (5.14, 17.00)^d,g^

Non-normally distributed data are presented as medians (first quartile, third quartile) and compared via nonparametric Kruskal–Wallis test. Categorical variables are expressed as numbers (%) and tested using chi-square test or Fisher’s test. *Post hoc* analyses were performed with the Bonferroni test. Different letters a, b, c, d, e, f, g and h represent significant differences between groups: *P* < 0.05.

CLBR, cumulative live birth rate; DOR, diminished ovarian reserve; OPU, oocyte pick-up; COH, controlled ovarian hyperstimulation; PPOS, progestin-primed ovarian stimulation; FET, frozen embryo transfer.

The CLBRs for Groups 1–9 over five FET cycles determined with the optimistic method (Fig. 3A1) were 91.7%, 93.1%, 96.6%, 79.2%, 89.9%, 76.1%, 90.0%, 92.9%, and 35.4%, respectively. With the conservative method (Fig. 3A2), the CLBRs were 77.3%, 79.4%, 88.9%, 46.7%, 72.7%, 62.1%, 74.4%, 78.8%, and 20.1% over five FET cycles. According to the multivariate regression analysis, the combined factors showed a decreasing trend for the aHR compared with that of the tubal factor infertility group according to the Cox model. According to the Fine–Gray model, the uterine factor and combined factors showed a decreasing trend for the aHR compared with the tubal factor infertility group.

## Discussion

This study revealed a significant attenuation in the association between an increase in the CLBR and the number of FET cycles with increasing female age. The CLBRs of Groups 1–5 gradually increased as the number of retrieved oocytes increased, irrespective of whether the optimistic or conservation method was employed. The causes of infertility, ranked in descending order of the CLBR, were as follows: PCOS, male factor, unexplained infertility, tubal factor, endometriosis, combined factors, uterine factor, DOR, and other infertility.

The negative impact of female age on ART treatment outcomes has been reported in other studies. The CLBR in the first complete cycle among 20 687 women who underwent FET significantly decreased with increasing age, reaching 63.8% for patients under 31 years of age compared with only 4.7% for patients over 40 years of age (Zhu *et al.*, 2018). Studies conducted by Liu *et al.* (2022) and Zhu *et al.* (2021) demonstrated that age was an independent risk factor for a low CLBR in women undergoing a single ovarian stimulation cycle as well as subsequent fresh and FETs. However, the optimal number of IVF attempts for older patients remains controversial.

Three small-scale retrospective studies have reported varying recommendations for the ideal number of IVF and fresh embryo transfer attempts: three attempts in patients aged 44–45 years (Raz *et al.*, 2018), four attempts in patients over 40 years of age (Khalife *et al.*, 2020), and six attempts in patients aged 41–44 years (Lebovitz *et al.*, 2018). On the basis of our extensive dataset, patients may consider oocyte retrieval and repeated embryo transfer until the age of 39 years. The CLBR consistently increased with each additional FET attempt. For patients aged 40–44 years, an optimistic approach allows for up to five or more attempts, whereas under a conservative approach, the CLBR tends to plateau after three attempts. Individuals older than 45 years are advised to discontinue treatment after three attempts, as there is a minimal further increase in the CLBR.

Several studies have investigated the correlation between the CLBR and the number of retrieved oocytes. A retrospective study involving 402 411 cycles (Fanton *et al.*, 2023) suggested that an increase in the number of retrieved oocytes is associated with a high CLBR. Some studies claim that the optimal number of oocytes required to achieve the maximum CLBR may vary with age. For individuals under 35 years of age, it is recommended that ∼25 oocytes be retrieved (Law *et al.*, 2019; Neves *et al.*, 2023). In our search, the CLBRs of Groups 1–5 demonstrated a consistent and progressive increase as the number of retrieved oocytes increased, regardless of whether the optimistic or conservative method was used.

Most studies have focused primarily on the influence of infertility aetiology on an individual’s reproductive outcomes. For example, Li *et al.* (2019) reported that patients diagnosed with PCOS, particularly patients aged over 40 years, presented significantly greater CLBRs than those diagnosed with tubal factor infertility. Another study revealed that the CLBR in Group 1, comprising patients with normal sperm and no tubo-peritoneal pathology on laparoscopy, was significantly lower than that in controls with severe male factor infertility, bilateral tubal block, and grade 3/4 endometriosis (Satwik and Kochhar, 2021). However, a comprehensive classification of the CLBR for different causes of infertility is currently lacking, and overall comparisons are not available. Our study first ranked the CLBR curves in descending order, with the PCOS group having the highest ranking, followed by the male factor, unexplained infertility, tubal factor, endometriosis, combined factors, uterine factor, DOR, and other infertility factors for both the optimistic and conservative methods. Women with DOR and other infertility factors have a lower chance of increasing their CLBR within five FET cycles and derive fewer benefits from repeated FETs.

Women aged over 45 years do not derive any benefits from repeated FETs after three attempts, and women aged 40–45 years or those with DOR and other infertility factors have a lower chance of improving their CLBR within five FET cycles. The results of the Cox and Fine-Gray multivariate analyses suggest that advanced age remains the primary factor impacting the CLBR. The data in Fig. 2A1 and A2 indicate that an advanced age >40 years might restrict the CLBR by up to 50%. Age is closely related to female reproductive ability, and 41 years of age is considered the age at which fertility stops and infertility starts (Tarlatzis and Zepiridis, 2003). Compared with that of young mice, the ovarian reserve function of old mice is severely weakened, as indicated by a reduced number of ovarian follicles, significantly decreased oocyte quality, significantly decreased oocyte mitochondrial membrane potential, a greater rate of abnormal spindle assembly after ICSI, and a significantly lower rate of fertilization and blastocyst formation (Tang *et al.*, 2020). In addition, studies have revealed that with increasing age, mitochondrial respiratory chain dysfunction, endoplasmic reticulum stress, and decreased antioxidant capacity lead to oocyte senescence (Zhang *et al.*, 2019).

The limitation of this study is that it was difficult to calculate the actual CLBR of each patient because some patients had remaining embryos that had not been transferred; additionally, the current statistical methodology utilizes both the optimistic and conservative methods to calculate the CLBR. The censoring in the Kaplan–Meier method/Cox model is considered non-informative, indicating that we assume that patients who discontinue treatment without achieving a live birth have an equal probability of experiencing a live birth in subsequent periods as those who are not censored (Clark *et al.*, 2003; Su *et al.*, 2022). This assumption may result in an overestimation of the true CLBR, with the actual curve likely to be lower. In contrast, within the Fine–Gray model, patients who discontinue treatment are categorized into two groups: those with no remaining embryos are counted as competing events, which tends to decrease the cumulative incidence of the outcome and represents a more pessimistic estimate (Fine and Gray, 1999), whereas those with remaining embryos but choose to discontinue treatment are considered censored. The Fine–Gray model adjusts for the impact of censored data by calculating weights based on probabilities of not experiencing either the primary event or competing event at the time of censoring, reflecting the risk of these events occurring in the absence of censoring (Wolbers *et al.*, 2014; Donoghoe and Gebski, 2017). These calculations are complex and can be automatically performed via R’s cmrpsk package. The cumulative incidence calculated by the Fine–Gray model may be lower than the actual rate. Therefore, in real-life scenarios, the CLBR falls between the optimistic and conservative curves, providing more comprehensive and objective information for clinical decision-making. Moreover, this study was based on data from a single centre; hopefully, there will be joint data from other centres in the future.

In conclusion, our study suggests that age, rather than the number of retrieved oocytes, is the primary factor that women should consider when deciding to discontinue FET treatment. Additionally, the cause of infertility should also be considered. For women over 45 years of age, discontinuation of treatment after three FET attempts is recommended since they do not derive any benefits from this approach. Women aged 40–45 years and those with DOR and other causes of infertility have a lower chance of improving their CLBR within five FET cycles and receive fewer advantages from repeated transfers. Furthermore, a combination of two or three infertility factors mentioned above will further decrease the age threshold at which repeated FET fails to yield any benefits.

## Supplementary Material

hoae063_Supplementary_Data

## Data Availability

The data underlying this article will be shared by the corresponding author upon reasonable request.

## References

[hoae063-B1] Abdullah RK , Liu N, Zhao Y, Shuang Y, Shen Z, Zeng H, Wu J. Cumulative live-birth, perinatal and obstetric outcomes for POSEIDON groups after IVF/ICSI cycles: a single-center retrospective study. Sci Rep 2020;10:11822. 32678263 10.1038/s41598-020-68896-1 PMC7366673

[hoae063-B05594938] Austin PC, , Lee DS, , Fine JP. Introduction to the Analysis of Survival Data in the Presence of Competing Risks. Circulation 2016;133:601–609. 26858290 10.1161/CIRCULATIONAHA.115.017719 PMC4741409

[hoae063-B2] Becker CM , Bokor A, Heikinheimo O, Horne A, Jansen F, Kiesel L, King K, Kvaskoff M, Nap A, Petersen K et al; ESHRE Endometriosis Guideline Group. ESHRE guideline: endometriosis. Hum Reprod Open 2022;2022:hoac009. 35350465 10.1093/hropen/hoac009 PMC8951218

[hoae063-B3] Chen H , Teng XM, Sun ZL, Yao D, Wang Z, Chen ZQ. Comparison of the cumulative live birth rates after 1 in vitro fertilization cycle in women using gonadotropin-releasing hormone antagonist protocol vs. progestin-primed ovarian stimulation: a propensity score–matched study. Fertil Steril 2022;118:701–712. 35940929 10.1016/j.fertnstert.2022.06.012

[hoae063-B4] Chen H , Wang Y, Lyu Q, Ai A, Fu Y, Tian H, Cai R, Hong Q, Chen Q, Shoham Z et al Comparison of live-birth defects after luteal-phase ovarian stimulation vs. conventional ovarian stimulation for in vitro fertilization and vitrified embryo transfer cycles. Fertil Steril 2015;103:1194–1201.e2. 25813280 10.1016/j.fertnstert.2015.02.020

[hoae063-B5] Clark TG , Bradburn MJ, Love SB, Altman DG. Survival analysis part I: basic concepts and first analyses. Br J Cancer 2003;89:232–238. 12865907 10.1038/sj.bjc.6601118 PMC2394262

[hoae063-B6] Cummins JM , Breen TM, Harrison KL, Shaw JM, Wilson LM, Hennessey JF. A formula for scoring human embryo growth rates in in vitro fertilization: its value in predicting pregnancy and in comparison with visual estimates of embryo quality. J In Vitro Fert Embryo Transf 1986;3:284–295. 10.1007/BF01133388 3783014

[hoae063-B7] Donoghoe MW , Gebski V. The importance of censoring in competing risks analysis of the subdistribution hazard. BMC Med Res Methodol 2017;17:52. 28376736 10.1186/s12874-017-0327-3 PMC5379776

[hoae063-B8] Fanton M , Cho JH, Baker VL, Loewke K. A higher number of oocytes retrieved is associated with an increase in fertilized oocytes, blastocysts, and cumulative live birth rates. Fertil Steril 2023;119:762–769. 36634732 10.1016/j.fertnstert.2023.01.001

[hoae063-B9] Fine JP , Gray RJ. A proportional hazards model for the subdistribution of a competing risk. J Am Stat Assoc 1999;94:496–509.

[hoae063-B10] Gardner DK , Simón C (eds). Handbook of In Vitro Fertilization. Boca Raton, FL, USA: CRC Press: Taylor & Francis Group, 2017.Available from: https://www.taylorfrancis.com/books/9781498729390 (18 July 2023, date last accessed).

[hoae063-B11] Guideline Group On Unexplained Infertility; Romualdi D, Ata B, Bhattacharya S, Bosch E, Costello M, Gersak K, Homburg R, Mincheva M, Norman RJ et al Evidence-based guideline: unexplained infertility. Hum Reprod 2023;38:1881–1890. 37599566 10.1093/humrep/dead150 PMC10546081

[hoae063-B12] Khalife D , Nassar A, Khalil A, Awwad J, Abu Musa A, Hannoun A, El Taha L, Khalifeh F, Abiad M, Ghazeeri G. Cumulative live-birth rates by maternal age after one or multiple in vitro fertilization cycles: an institutional experience. Int J Fertil Steril 2020;14:34–40. 32112633 10.22074/ijfs.2020.5855 PMC7139227

[hoae063-B13] Kuang Y , Chen Q, Fu Y, Wang Y, Hong Q, Lyu Q, Ai A, Shoham Z. Medroxyprogesterone acetate is an effective oral alternative for preventing premature luteinizing hormone surges in women undergoing controlled ovarian hyperstimulation for in vitro fertilization. Fertil Steril 2015;104:62–70.e3. 25956370 10.1016/j.fertnstert.2015.03.022

[hoae063-B14] Kuang Y , Chen Q, Hong Q, Lyu Q, Ai A, Fu Y, Shoham Z. Double stimulations during the follicular and luteal phases of poor responders in IVF/ICSI programmes (Shanghai protocol). Reprod Biomed Online2014a;29:684–691. 25444501 10.1016/j.rbmo.2014.08.009

[hoae063-B15] Kuang Y , Hong Q, Chen Q, Lyu Q, Ai A, Fu Y, Shoham Z. Luteal-phase ovarian stimulation is feasible for producing competent oocytes in women undergoing in vitro fertilization/intracytoplasmic sperm injection treatment, with optimal pregnancy outcomes in frozen-thawed embryo transfer cycles. Fertil Steril2014b;101:105–111. 24161646 10.1016/j.fertnstert.2013.09.007

[hoae063-B16] Kundu D , Kannan N, Balakrishnan N. Analysis of progressively censored competing risks data. In: N. Balakrishnan N, Rao CR (eds). *Handbook of Statistics.* Vol. 23, Amsterdam, The Netherlands: Elsevier, 2003, 331–348.

[hoae063-B17] Law YJ , Zhang N, Venetis CA, Chambers GM, Harris K. The number of oocytes associated with maximum cumulative live birth rates per aspiration depends on female age: a population study of 221 221 treatment cycles. Hum Reprod 2019;34:1778–1787. 31398253 10.1093/humrep/dez100

[hoae063-B18] Lebovitz O , Haas J, James KE, Seidman DS, Orvieto R, Hourvitz A. The expected cumulative incidence of live birth for patients starting IVF treatment at age 41 years or older. Reprod Biomed Online 2018;37:533–541. 30297113 10.1016/j.rbmo.2018.08.014

[hoae063-B19] Li J , Liu X, Hu L, Zhang F, Wang F, Kong H, Dai S, Guo Y. A Slower age-related decline in treatment outcomes after the first ovarian stimulation for in vitro fertilization in women with polycystic ovary syndrome. Front Endocrinol (Lausanne) 2019;10:834. 31866942 10.3389/fendo.2019.00834 PMC6906164

[hoae063-B20] Liu M , Zhao X, Peng Y, Zheng J, Guo K, Fan Y, Jiang L, Yang A, Cui N, Hao G et al Outcomes after a single ovarian stimulation cycle in women of advanced reproductive age: a retrospective analysis. Front Endocrinol (Lausanne) 2022;13:792159. 35237234 10.3389/fendo.2022.792159 PMC8882593

[hoae063-B21] Maheshwari A , McLernon D, Bhattacharya S. Cumulative live birth rate: time for a consensus? Hum Reprod 2015;30:2703–2707. 26466912 10.1093/humrep/dev263

[hoae063-B22] McLernon DJ , Maheshwari A, Lee AJ, Bhattacharya S. Cumulative live birth rates after one or more complete cycles of IVF: a population-based study of linked cycle data from 178,898 women. Hum Reprod 2016;31:572–581. 26783243 10.1093/humrep/dev336

[hoae063-B23] Neves AR , Montoya-Botero P, Sachs-Guedj N, Polyzos NP. Association between the number of oocytes and cumulative live birth rate: a systematic review. Best Pract Res Clin Obstet Gynaecol 2023;87:102307. 36707342 10.1016/j.bpobgyn.2022.102307

[hoae063-B1046574] Pastore LM, , Christianson MS, , Stelling J, , Kearns WG, , Segars JH. Reproductive ovarian testing and the alphabet soup of diagnoses: DOR, POI, POF, POR, and FOR. J Assist Reprod Genet 2018;35:17–23. 28971280 10.1007/s10815-017-1058-4 PMC5758472

[hoae063-B0506307] Penzias A, , Azziz R, , Bendikson K, , Falcone T, , Hansen K, , Hill M, , Hurd W, , Jindal S, , Kalra S, , Mersereau J et al Testing and interpreting measures of ovarian reserve: a committee opinion. Fertility and Sterility 2020;114:1151–1157. 33280722 10.1016/j.fertnstert.2020.09.134

[hoae063-B24] Qin N , Chen Q, Hong Q, Cai R, Gao H, Wang Y, Sun L, Zhang S, Guo H, Fu Y et al Flexibility in starting ovarian stimulation at different phases of the menstrual cycle for treatment of infertile women with the use of in vitro fertilization or intracytoplasmic sperm injection. Fertil Steril 2016;106:334–341.e1. 27114329 10.1016/j.fertnstert.2016.04.006

[hoae063-B25] Raz N , Shalev A, Horowitz E, Weissman A, Mizrachi Y, Ganer Herman H, Raziel A. Cumulative pregnancy and live birth rates through assisted reproduction in women 44–45 years of age: is there any hope? J Assist Reprod Genet 2018;35:441–447. 29218446 10.1007/s10815-017-1094-0 PMC5904064

[hoae063-B26] Saket Z , Källén K, Lundin K, Magnusson Å, Bergh C. Cumulative live birth rate after IVF: trend over time and the impact of blastocyst culture and vitrification. Hum Reprod Open 2021;2021:hoab021. 34195386 10.1093/hropen/hoab021 PMC8240131

[hoae063-B27] Satwik R , Kochhar M. Unexplained infertility categorization based on female laparoscopy and total motile sperm count, and its impact on cumulative live-births after one in-vitro fertilization cycle. A retrospective cohort study involving 721 cycles. Reprod Med Biol 2021;20:190–198. 33850452 10.1002/rmb2.12368 PMC8022093

[hoae063-B28] Shen X , Xie Y, Chen D, Guo W, Feng G, Jiang W, Long H, Lyu Q, Jin W, Kuang Y et al Effect of female and male body mass index on cumulative live birth rates in the freeze-all strategy. J Clin Endocrinol Metab 2022;107:e1467–e1476. 34850010 10.1210/clinem/dgab858

[hoae063-B29] Stolwijk AM , Hamilton CJ, Hollanders JM, Bastiaans LA, Zielhuis GA. A more realistic approach to the cumulative pregnancy rate after in-vitro fertilization. Hum Reprod 1996;11:660–663. 8671287 10.1093/humrep/11.3.660

[hoae063-B30] Su P-F , Lin C-CK, Hung J-Y, Lee J-S. The proper use and reporting of survival analysis and cox regression. World Neurosurg 2022;161:303–309. 35505548 10.1016/j.wneu.2021.06.132

[hoae063-B5824572] Tal R, , Seifer DB. Ovarian reserve testing: a user's guide. Am J Obstet Gynecol 2017;217:129–140. 28235465 10.1016/j.ajog.2017.02.027

[hoae063-B31] Tang M , Popovic M, Stamatiadis P, Van Der Jeught M, Van Coster R, Deforce D, De Sutter P, Coucke P, Menten B, Stoop D et al Germline nuclear transfer in mice may rescue poor embryo development associated with advanced maternal age and early embryo arrest. Hum Reprod 2020;35:1562–1577. 32613230 10.1093/humrep/deaa112

[hoae063-B32] Tarlatzis BC , Zepiridis L. Perimenopausal conception. Ann New York Acad Sci 2003;997:93–104. 14644814 10.1196/annals.1290.011

[hoae063-B33] Teede HJ , Misso ML, Costello MF, Dokras A, Laven J, Moran L, Piltonen T, Norman RobertJ Andersen M, Azziz R; International PCOS Network. Recommendations from the international evidence-based guideline for the assessment and management of polycystic ovary syndrome. Fertil Steril 2018;110:364–379. 30033227 10.1016/j.fertnstert.2018.05.004 PMC6939856

[hoae063-B34] Toftager M , Bogstad J, Løssl K, Prætorius L, Zedeler A, Bryndorf T, Nilas L, Pinborg A. Cumulative live birth rates after one ART cycle including all subsequent frozen–thaw cycles in 1050 women: secondary outcome of an RCT comparing GnRH-antagonist and GnRH-agonist protocols. Hum Reprod 2017;32:556–567. 28130435 10.1093/humrep/dew358

[hoae063-B35] Wang Z , Groen H, Van Zomeren KC, Cantineau AEP, Van Oers A, Van Montfoort APA, Kuchenbecker WKH, Pelinck MJ, Broekmans FJM, Klijn NF et al Lifestyle intervention prior to IVF does not improve embryo utilization rate and cumulative live birth rate in women with obesity: a nested cohort study. Hum Reprod Open 2021;2021:hoab032. 34557597 10.1093/hropen/hoab032 PMC8452483

[hoae063-B36] Wilkinson J , Roberts SA, Showell M, Brison DR, Vail A. No common denominator: a review of outcome measures in IVF RCTs. Hum Reprod 2016;31:2714–2722. 27664214 10.1093/humrep/dew227 PMC5193327

[hoae063-B37] Wolbers M , Koller MT, Stel VS, Schaer B, Jager KJ, Leffondré K, Heinze G. Competing risks analyses: objectives and approaches. Eur Heart J 2014;35:2936–2941. 24711436 10.1093/eurheartj/ehu131 PMC4223609

[hoae063-B38] Zhang S , Yin Y, Li Q, Zhang C. Comparison of cumulative live birth rates between GnRH-A and PPOS in low-prognosis patients according to POSEIDON criteria: a cohort study. Front Endocrinol (Lausanne) 2021;12:644456. 34234739 10.3389/fendo.2021.644456 PMC8256850

[hoae063-B39] Zhang T , Xi Q, Wang D, Li J, Wang M, Li D, Zhu L, Jin L. Mitochondrial dysfunction and endoplasmic reticulum stress involved in oocyte aging: an analysis using single-cell RNA-sequencing of mouse oocytes. J Ovarian Res 2019;12:53. 31176373 10.1186/s13048-019-0529-x PMC6556043

[hoae063-B40] Zhu H , Zhao C, Xiao P, Zhang S. Predicting the likelihood of live birth in assisted reproductive technology according to the number of oocytes retrieved and female age using a generalized additive model: a retrospective cohort analysis of 17,948 cycles. Front Endocrinol (Lausanne) 2021;12:606231. 33995268 10.3389/fendo.2021.606231 PMC8120808

[hoae063-B41] Zhu Q , Chen Q, Wang L, Lu X, Lyu Q, Wang Y, Kuang Y. Live birth rates in the first complete IVF cycle among 20 687 women using a freeze-all strategy. Hum Reprod 2018;33:924–929. 29534178 10.1093/humrep/dey044

