# Supplementary material for: Women may not benefit from repeated frozen embryo transfers: a retrospective analysis of the cumulative live birth rate of 43 972 women
Source: Hum Reprod Open. 2024 Oct 28;2024(4):hoae063. doi: 10.1093/hropen/hoae063 (PMC11557905; doi:10.1093/hropen/hoae063)
Supplement: hoae063_Supplementary_Data [file hoae063_supplementary_data.zip › Supplementary figure S1-rev.pdf]

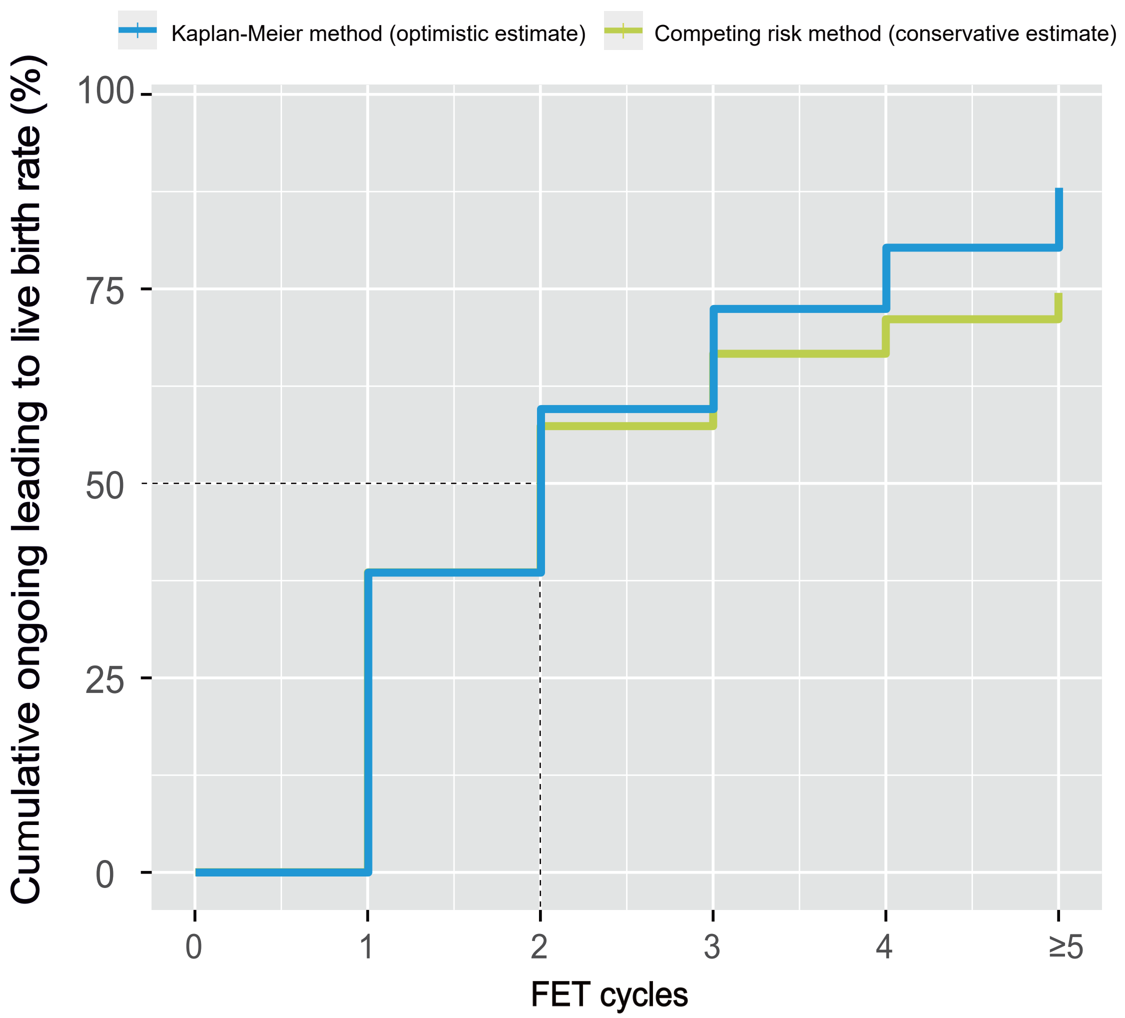

Supplementary figure S1. Cumulative live birth curves for 43,972 patients who underwent frozen embryo transfer cycles using the Kaplan–Meier method (optimistic method; blue) and the competing risk method (conservative method; green). FET: frozen-thawed embryo transfer.
