# Supplementary material for: Women may not benefit from repeated frozen embryo transfers: a retrospective analysis of the cumulative live birth rate of 43 972 women
Source: Hum Reprod Open. 2024 Oct 28;2024(4):hoae063. doi: 10.1093/hropen/hoae063 (PMC11557905; doi:10.1093/hropen/hoae063)
Supplement: hoae063_Supplementary_Data [file hoae063_supplementary_data.zip › New-Supplementary-Tables-post adjudication clean.docx]

**Supplementary Table S1.** The single-variable and multivariable analysis of potential confounding factors affecting the CLBR of patients under COX model.

| **Confounding factors** | **HR (95%CI)** | **P value** | **Adjusted HR (95%CI)** | **P value** |
| --- | --- | --- | --- | --- |
| **Duration of infertility** | 0.98 (0.97,0.98) | <0.001 | 0.99 (0.99,0.990 | 0.002 |
| **Infertility type** |  | <0.001 |  | <0.001 |
| Primary infertility | reference |  | reference |  |
| Secondary infertility | 0.85 (0.83,0.87) | <0.001 | 0.95 (0.92,0.97) | <0.001 |
| **Basal FSH (IU/L)** |  | <0.001 |  | 0.673 |
| <10 | reference |  | reference |  |
| 10-15 | 0.64 (0.59,0.69) | <0.001 | 0.99 (0.92,1.07) | 0.808 |
| >15 | 0.57 (0.49,0.65) | <0.001 | 0.94 (0.81,1.09) | 0.384 |
| **Antral Follicle Counts (n)** |  | <0.001 |  | 0.321 |
| 0-5 | reference |  | reference |  |
| 6-10 | 1.23 (1.19,1.27) | <0.001 | 1.03 (0.99,1.07) | 0.135 |
| 10-15 | 1.38 (1.33,1.43) | <0.001 | 1.03 (0.99,1.07) | 0.202 |
| 15-20 | 1.48 (1.42,1.53) | <0.001 | 1.04 (1.00,1.09) | 0.050 |
| >20 | 1.50 (1.42,1.57) | <0.001 | 1.05 (1.00,1.11) | 0.089 |
| **Male age (year)** |  | <0.001 |  | 0.071 |
| <30 | reference |  | reference |  |
| 30-34 | 0.91 (0.88,0.93) | <0.001 | 0.98 (0.95,1.01) | 0.204 |
| 35-39 | 0.79 (0.76,0.82) | <0.001 | 0.97 (0.93,1.01) | 0.152 |
| 40-44 | 0.54 (0.51,0.56) | <0.001 | 0.91 (0.86,0.97) | 0.004 |
| ≥45 | 0.38 (0.35,0.41) | <0.001 | 0.94 (0.86,1.02) | 0.157 |
| **Female BMI (kg/m^2^)** |  | <0.001 |  | 0.008 |
| <18.5 | reference |  | reference |  |
| 18.5-23.99 | 0.92 (0.89,0.96) | <0.001 | 0.99 (0.95,1.03) | 0.486 |
| 24.0-27.99 | 0.84 (0.80,0.88) | <0.001 | 0.95 (0.90,0.99) | 0.022 |
| ≥28 | 0.85 (0.80,0.91) | <0.001 | 0.92 (0.86,0.98) | 0.012 |
| **OPU times (n)** | 0.75 (0.74,0.76) | <0.001 | 0.90 (0.89,0.91) | <0.001 |
| **FET times (n)** | 0.62 (0.61,0.62) | <0.001 | 0.65 (0.63,0.68) | <0.001 |
| **Total oocyte number (n)** | 0.99 (0.99,0.99) | <0.001 | 1.00 (1.00,1.00) | 0.353 |
| **Total embryo number (n)** | 0.96 (0.96,0.96) | <0.001 | 1.04 (1.03,1.04) | <0.001 |
| **Number of embryos transferred (n)** | 0.79 (0.79,0.80) | <0.001 | 0.99 (0.96,1.02) | 0.494 |
| **Number of good-quality embryos transferred (n)** | 0.81 (0.81,0.82) | <0.001 | 0.97 (0.95,0.98) | <0.001 |
| **Patients’ first OPU year** |  | <0.001 |  | <0.001 |
| 2010-2013 | reference |  | reference |  |
| 2014-2015 | 0.99 (0.96,1.03) | 0.682 | 0.89 (0.86,0.92) | <0.001 |
| 2016-2017 | 0.91 (0.88,0.95) | <0.001 | 0.66 (0.64,0.69) | <0.001 |
| 2018-2019 | 0.86 (0.83,0.90) | <0.001 | 0.62 (0.60,0.65) | <0.001 |
| 2020-2023 | 0.58 (0.55,0.61) | <0.001 | 0.48 (0.45,0.50) | <0.001 |

P < 0.05 was considered statistically significant. CLBR, cumulative live birth rate; HR, hazard ratio; OPU, oocyte pick-up; FET, frozen embryo transfer.

**Supplementary Table S2**. The single-variable and multivariable analysis of potential confounding factors affecting the CLBR of patients under Fine-Gray model.

| **Confounding factors** | **HR (95%CI)** | **P value** | **Adjusted HR (95%CI)** | **P value** |
| --- | --- | --- | --- | --- |
| **Duration of infertility** | 0.97 (0.97,0.97) | <0.001 | 0.99 (0.98,0.99) | <0.001 |
| **Infertility type** |  | <0.001 |  |  |
| Primary infertility | reference |  | reference |  |
| Secondary infertility | 0.82 (0.80,0.84) | <0.001 | 0.93 (0.92,0.95) | <0.001 |
| **Basal FSH (IU/L)** |  | <0.001 |  | 0.023 |
| <10 | reference |  | reference |  |
| 10-15 | 0.54 (0.50,0.58) | <0.001 | 0.93 (0.87,0.99) | 0.036 |
| >15 | 0.43 (0.37,0.49) | <0.001 | 0.83 (0.72,0.96) | 0.011 |
| **Antral Follicle Counts (n)** |  | <0.001 |  | <0.001 |
| 0-5 | reference |  | reference |  |
| 6-10 | 1.36 (1.32,1.40) | <0.001 | 1.06 (1.03,1.09) | <0.001 |
| 10-15 | 1.61 (1.56,1.66) | <0.001 | 1.08 (1.04,1.11) | <0.001 |
| 15-20 | 1.76 (1.71,1.82) | <0.001 | 1.08 (1.05,1.12) | <0.001 |
| >20 | 1.78 (1.71,1.85) | <0.001 | 1.08 (1.04,1.12) | <0.001 |
| **Male age (year)** |  | <0.001 |  | <0.001 |
| <30 | reference |  | reference |  |
| 30-34 | 0.89 (0.87,0.91) | <0.001 | 0.98 (0.96,1.01) | 0.14 |
| 35-39 | 0.74 (0.72,0.76) | <0.001 | 0.97 (0.94,1.01) | 0.11 |
| 40-44 | 0.47 (0.45,0.49) | <0.001 | 0.90 (0.86,0.95) | <0.001 |
| ≥45 | 0.31 (0.29,0.34) | <0.001 | 0.95 (0.88,1.01) | 0.12 |
| **Female BMI (kg/m^2^)** |  | <0.001 |  | <0.001 |
| <18.5 | reference |  | reference |  |
| 18.5-23.99 | 0.91 (0.88,0.94) | <0.001 | 1.00 (0.97,1.03) | 0.91 |
| 24.0-27.99 | 0.82 (0.78,0.85) | <0.001 | 0.95 (0.92,0.99) | 0.01 |
| ≥28 | 0.84 (0.79,0.88) | <0.001 | 0.93 (0.89,0.99) | 0.012 |
| **OPU times (n)** | 0.75 (0.74,0.76) | <0.001 | 0.90 (0.89,0.91) | <0.001 |
| **FET times (n)** | 0.72 (0.72,0.73) | <0.001 | 0.66 (0.64,0.69) | <0.001 |
| **Total oocyte number (n)** | 0.99 (0.99,1.00) | 0.003 | 0.99 (0.99,0.99) | <0.001 |
| **Total embryo number (n)** | 1.02 (1.02,1.02) | <0.001 | 1.08 (1.08,1.09) | <0.001 |
| **Number of embryos transferred (n)** | 0.87 (0.87,0.88) | <0.001 | 1.02 (0.99,1.04) | 0.12 |
| **Number of good-quality embryos transferred (n)** | 0.88 (0.87,0.88) | <0.001 | 0.97 (0.96,0.98) | <0.001 |
| **Patients’ first OPU year** |  | <0.001 |  | <0.001 |
| 2010-2013 | reference |  | reference |  |
| 2014-2015 | 1.02 (0.99,1.04) | 0.23 | 1.00 (0.97,1.02) | 0.90 |
| 2016-2017 | 0.91 (0.88,0.93) | <0.001 | 0.79 (0.77,0.82) | <0.001 |
| 2018-2019 | 0.90 (0.87,0.93) | <0.001 | 0.78 (0.75,0.80) | <0.001 |
| 2020-2023 | 0.55 (0.53,0.58) | <0.001 | 0.49 (0.47,0.51) | <0.001 |

P < 0.05 was considered statistically significant. CLBR, cumulative live birth rate; HR, hazard ratio; OPU, oocyte pick-up; FET, frozen embryo transfer.

**Supplementary Table S3**. The detailed cumulative live birth rate and censoring data across treatment cycles for the entire cohort of 43,972 patients using Kaplan-Meier method.

| **Kaplan-Meier method** | |  |  |  |  |
| --- | --- | --- | --- | --- | --- |
| **FET cycle**  **Number**  **(Time)** | **Number of women who underwent treatment (Risk group)** | **Number of live births**  **(Events)** | **Number of women who stopped treatment (Censoring)** | **Probability of not having live birth up to the cycle** | **Cumulative incidence of live birth up to the cycle** |
| 1st | 43972 | 16926 | 6488 | 0.615 | 0.385 |
| 2nd | 20558 | 7018 | 5153 | 0.405 | 0.595 |
| 3rd | 8387 | 2661 | 2366 | 0.277 | 0.723 |
| 4th | 3360 | 955 | 1035 | 0.198 | 0.802 |
| ≥5th | 1370 | 532 | 838 | 0.121 | 0.879 |

FET, frozen embryo transfer.

**Supplementary Table S4**. The detailed cumulative live birth rate, censoring data and competing events across treatment cycles for the entire cohort of 43,972 patients using Fine-Gray model.

| **Fine-Gray model** | |  |  |  |  |  |
| --- | --- | --- | --- | --- | --- | --- |
| **FET cycle**  **Number**  **(Time)** | **Number of women who underwent treatment (Risk group)** | **Number of live births**  **(Events)** | **Number of women who used all her embryos (competing events)** | **Number of women who stopped treatment (Censoring)** | **Probability of not having live birth up to the cycle** | **Cumulative incidence of live birth up to the cycle** |
| 1st | 43972 | 16926 | 2877 | 3611 | 0.615 | 0.385 |
| 2nd | 20558 | 7018 | 2585 | 2568 | 0.427 | 0.573 |
| 3rd | 8387 | 2661 | 1266 | 1100 | 0.335 | 0.665 |
| 4th | 3360 | 955 | 523 | 512 | 0.290 | 0.710 |
| ≥5th | 1370 | 532 | 415 | 423 | 0.256 | 0.744 |

FET, frozen embryo transfer.
